# Dynamic integrin expression, atypical nuclear localization, and spatial distribution during ovarian cancer progression and metastasis

**DOI:** 10.3389/fcell.2026.1744403

**Published:** 2026-02-25

**Authors:** Nazia Bano, Jack L. Browning, Isabelle Lewis, Malaika Amin, Piper Zimmerman, Lina Lee, Eva M. Schmelz

**Affiliations:** 1 Translational Biology, Medicine and Health, Virginia Tech, Blacksburg, VA, United States; 2 School of Neuroscience, Virginia Tech, Blacksburg, VA, United States; 3 Department of Human Nutrition, Foods and Exercise, Virginia Tech, Blacksburg, VA, United States; 4 Department of Biology, Hollins University, Roanoke, VA, United States; 5 Department of Statistics, Virginia Tech, Blacksburg, VA, United States

**Keywords:** adhesion, ECM, integrins, metastasis, nuclear localization, ovarian cancer, progression, slow developing and aggressive disease

## Abstract

**Introduction:**

Aggregation and adhesion of ovarian cancer cells are facilitated by integrins, key adhesion receptors in ovarian cancer. Here we identify changes in the expression of integrins (ITG), their ligands and regulators during ovarian cancer progression and metastatic dissemination that allow for the adaptation of the cellular phenotype to aggregation and adhesion and promote cancer cell survival and metastatic outgrowth.

**Methods:**

We mimicked the stages of peritoneal dissemination of ovarian metastases by using benign cells and ovarian cancer cells representing slow- and fast developing disease and generated adherent, spheroid, and adherent-spheroid mouse ovarian surface epithelial cultures adjusted for oxygen and glucose levels as reported for malignant ascites. We determined changes in integrin expression, other adhesion receptors, ECM proteins and their regulators by qPCR RT2 PCR arrays and Western blotting. Spatial and intracellular protein expression in 3D spheroids was determined by confocal microscopy and quantitated by IMARIS software. Relevance of specific integrins for aggregation, adherence, and outgrowth was determined using specific inhibitors.

**Results:**

Small changes in the highly expressed *ITGα3, ITGα5, ITGαV*, and *ITGβ1* after aggregation in concert with elevated ITGα4 and ITGα5 expression suggested changes in integrin heterodimer composition that support aggregation. 3D spatial analysis of adherent spheroids revealed high expression of ITGα2, ITGαV, and ITGβ1 at the adhesion sites, while ITGα3 was predominantly expressed in the spheroid periphery. This was not correlated to their distinct spatial expression patterns in spheroids (uniformly expressed or higher at the periphery). Importantly, most integrins and CD44 were localized in the nucleus where they potentially can affect gene transcription. Only the inhibition of ITGαVβ1 and ITGα2β1 effectively suppressed spheroid adhesion and outgrowth, highlighting their importance as stage-specific target to block peritoneal metastasis.

**Discussion:**

Our studies show that integrin expression and localization are dynamic, spatially regulated, and functionally compartmentalized during ovarian cancer progression and dissemination. The coordinated upregulation of integrins, other adhesion molecules (CD44, NCAM1, VCAM), ECM (FN1, collagens) and their regulators (SPP1, TIMP2,3) in response to the culture conditions indicate a complex reprogramming of adhesion networks that can facilitate different steps of ovarian cancer progression and dissemination. Nuclear localization of integrins and CD44 point to dual roles in adhesion, survival, and proliferation by activating adhesion-mediated signaling pathways and directly affect gene transcription that support a switch from a more dormant phenotype to active proliferation and invasion after adhesion.

## Introduction

During metastasis, ovarian cancer cells exfoliate from the primary tumor, are transported throughout the peritoneal cavity by the flow of peritoneal fluids or ascites and rapidly adhere to the omentum or other secondary sites (Farsinejad et al., 2019). This transcoelomic dissemination is associated with a 5-year survival rate of only 30.8% (Society, 2025). Current multistep models of ovarian metastasis suggest that ovarian metastasis begins by detachment of single cells from the tumor, followed by aggregation of the cells in the peritoneal cavity and attachment at distant sites and formation of secondary lesions (Lengyel, 2010). More recent reports also show a detachment of multicellular cell clusters (Al Habyan et al., 2018). In patients, both single cells and spheroids have been observed (Davidson, 2001; Latifi et al., 2012). Cancer spheroids are more resistant to drug treatment (Yoshida et al., 2008; Compton et al., 2021; Swierczewska et al., 2023) and apoptosis (Aceto et al., 2014; Cai et al., 2015) and readily form secondary lesions (Latifi et al., 2012). Immune and stromal cells can be recruited to the spheroids (Compton et al., 2021; Ding et al., 2021; Yin et al., 2016), further increasing the spheroids’ metastatic potential (Compton et al., 2021; Sodek et al., 2009; Motohara et al., 2019). Thus, ovarian spheroids disseminating in the peritoneal cavity are considered the main metastatic units. The increased survival potential of spheroids (Al Habyan et al., 2018) indicates that aggregation of metastases provides signals conducive to the promotion of tumor cell survival, causing the formation of secondary lesions and disease recurrence. These changes are not unique to ovarian cancer but have also been observed in colon, breast, glioma, and prostate cancer (Aceto et al., 2014; Rayavarapu et al., 2015). The underlying mechanisms of the increased survival potential have yet to be fully elucidated. Our previous studies indicate that aggregation of ovarian cancer cells caused an increase in anti-apoptotic, stemness, and angiogenesis-related genes while genes involved in metabolism and proliferation were decreased (Grieco et al., 2023a). This correlated well with a significantly reduced cellular metabolism and ATP synthesis, the fragmentation of the mitochondrial network and the subsequently reduced spheroid growth (Compton et al., 2021; Grieco et al., 2021). After adhesion of the spheroids, the successful outgrowth would require the reversal of the spheroidal phenotype. Indeed, we have shown a rapid outgrowth of the spheroidal cells after adhesion that was accompanied by an increased mitobiogenesis and glucose uptake (Compton et al., 2022; Grieco et al., 2023b). These changes were only observed at sites of adhesion but not in the bulk of the spheroids, suggesting that adhesion provides signals for a phenotypical adaptation of the cancer cells to their changing microenvironment.

Both cell-cell and cell-ECM play crucial roles in the aggregation of exfoliated ovarian cancer cells, their attachment to the mesothelial lining of the peritoneal cavity as well as their disaggregation and invasion into the extracellular matrix (ECM) of the peritoneum (Farsinejad et al., 2019; Shield et al., 2007). Integrins are recognized as important cell adhesion receptors in ovarian cancer. They are transmembrane glycoproteins consisting of 18 α subunits and 8 β subunits that combine in a non-covalent manner to form 24 distinct αβ heterodimer receptor complexes. The α integrin subunit primarily interacts with extracellular matrix (ECM) ligands from outside the cells, whereas the β subunit binds to actin cytoskeleton from inside the cells. These receptor complexes act as signaling hubs regulating both outside-in and inside-out signaling. Thus, the integrin heterodimer expression and composition and the ECM present in the tumor microenvironment will determine which signaling pathway is activated and, thereby, contribute to a distinct cellular response. In general, the interaction of integrin heterodimers with the ECM proteins stimulates proliferation and progression (see reviews (Cooper and Giancotti, 2019; Li et al., 2023; Hynes et al., 2002).

Several integrin (ITG) heterodimers have been shown to be involved in multiple stages of the metastatic process including the epithelial-mesenchymal transition, aggregation and adhesion of ovarian cancer cells, and disintegration and invasion the underlying matrix (see review (Dhaliwal and Shepherd, 2022)). Specifically, heterodimers including the β1 subunit were shown to be critical at different stages of metastasis. ITGα5β1 mediated the aggregation of the human ovarian cancer cell line OVCAR5, but not SKOV3 cells while other α subunits or CD44 had no effect. Other reports show the importance of ITGα5β1 for spheroid survival, adhesion to mesothelial cells, and invasion of the underlying ECM *in vitro* (Masi et al., 2023; Strobel and Cannistra, 1999) and both initial attachment and invasion of intraperitoneally injected SKOV3 cells (Mitra et al., 2011). ITGα2β1 has been implicated in spheroid disaggregation and proteolysis, key steps in spheroids adhesion and invasion at secondary sites (Shield et al., 2007). The high expression ITGα2β1 in ovarian tumors was associated with a lower progression-free survival (Dotzer et al., 2021). In platinum-resistant ovarian cancer cell lines, a high ITGα6 expression led to an increased adhesion to laminins, invasion of Matrigel *in vitro* and tumor growth in the peritoneal cavity (Gambelli et al., 2024). In contrast, while ITGαVβ3 contributed to ovarian cancer cell adhesion, it slowed tumor progression and may act as a tumor suppressor (Moser et al., 1996). These studies indicate that there is an intricate link between integrin expression, heterodimer and ECM composition, and activation of signaling pathways that determine the cells’ functions such as changing to a contractile morphology (Masi et al., 2023). However, the expression, combination, and function of the integrin heterodimers appear to be distinct in different cell lines as well as dependent on tissue culture conditions *in vitro*, and the stage of the disease and tissue origin *in vivo*.

While critically important, little is known about how integrin expression changes at different stages of ovarian cancer and the consequences of these changes on progression and dissemination, adhesion and secondary outgrowth. Using a progressive model of ovarian cancer mouse ovarian surface epithelium cells, here we characterize changes in integrin expression during spheroid formation and adhesion of ovarian cancer spheroids to secondary sites using *in vitro* culture conditions that better represent the *in vivo* oxygen and glucose conditions. These culture conditions mimic the transition from a normoxic, high-glucose environment at the primary tumor site to a hypoxic, low-glucose state within the peritoneal cavity–conditions that have been shown to selectively alter integrin expression in various cancers (Ju et al., 2017; Koivunen et al., 2025; Nam et al., 2024). Upon attachment to the mesothelium, the cancer cells re-enter a normoxic, high-glucose environment after gaining access to blood vessels and adapt their metabolic phenotype (Compton et al., 2021; Grieco et al., 2021; Compton et al., 2022; Grieco et al., 2023b) to ensure survival and successful metastasis.

Identifying the stage-dependent expression of integrins provides therapeutic targets for patients who present with advanced-stage disease characterized by widespread peritoneal dissemination and develop strategies targeting integrin signaling that may be especially effective in late-stage cancer. Such approaches could prevent secondary tumor outgrowth, reduce metastatic spread, and ultimately lower recurrence rates, which are currently a major cause of mortality in ovarian cancer patients.

## Materials and methods

### Cell culture

The mouse ovarian surface epithelium model represents the progression of ovarian cancer, encompassing benign (MOSE-E), slow-developing (MOSE-L), and fast-developing disease (MOSE-L_TIC*v*_) (Roberts et al., 2005). Their genotype and phenotype have been extensively characterized, and the expression of fallopian tube markers suggest that the MOSE cells represent serous ovarian cancer development (Creekmore et al., 2011; Cohen et al., 2013; Cohen et al., 2016; Anderson et al., 2013). MOSE-_TIC*v*_ cells were derived by harvesting the peritoneal fluid from mice injected with MOSE-L cells. This *in vivo* selection significantly increased the tumorigenicity from 1 × 10^6^ cells injected intraperitoneally into C57BL/6 mice causing lethal disease in ∼100 days (MOSE-L) to 1 × 104 cells causing lethal disease in 21 days (MOSE-L_TIC*v*_) (Roberts et al., 2005; Cohen et al., 2013). MOSE cells were routinely cultured in high-glucose DMEM (HG; 25 mM glucose) (Sigma-Aldrich), supplemented with 4% fetal bovine serum (FBS, Hyclone), 3.7 g/L sodium bicarbonate (Sigma-Aldrich), and 1% penicillin -streptomycin solution (Thermo Fisher Scientific) at 5% CO_2_ and 21% O_2_ (normoxia, NO). Spheroid formation was achieved as described (Compton et al., 2021; Compton et al., 2022). Briefly, cells were seeded into ultra-low adherent 96-well plates (Corning) in hypoxia (HO, 2% oxygen), and low-glucose (LG, 5 mM glucose) DMEM for 24 h. This represents the oxygen (Kizaka-Kondoh et al., 2009; Emoto et al., 2010; Mutch and Williams, 1994) and glucose levels (Lippi et al., 2013) found in malignant ascites. The benign MOSE-E were not used here because these cells do not form viable spheroids. To mimic spheroid adhesion to the secondary site, MOSE-L, and MOSE-L_TIC*V*_ spheroids were generated in a LG DMEM in HY for 24 h. To mimic initial attachment at secondary sites, spheroids were transferred to tissue culture-treated plates and kept in LG, HO conditions for 12 h. At this timepoint, the spheroids were attached to the tissue culture plates, slightly flattened and had begun to grow onto the culture plates (outgrowth). Gained access to oxygen and nutrients after adhesion and angiogenesis (reoxygenation) was mimicked by changing the culture conditions to HG medium with 4% FBS at NO for an additional 16 h. Thus, for the subsequent gene and protein analyses, the samples named ‘adherent spheroids’ contained both actively outgrowing cells and the bulk of the spheroid.

### Reverse transcriptase polymerase chain reaction

Total RNA was isolated from adherent cells, spheroids, and adherent-spheroids cultured in the conditions described above using the RNeasy Mini Kit (Qiagen). Complementary DNA (cDNA) was synthesized using the RT^2^ First Strand Kit (Qiagen), according to the manufacturer’s specifications. Gene expression profiling of adhesion molecules, their regulators, and extracellular matrix was performed using the RT^2^ Profiler PCR Array for mouse extracellular matrix and adhesion molecules (Cat. No. 330231 PAMM-013ZR, Qiagen; gene list in Supplementary Table S1). MOSE-L and MOSE-L_TIC*V*_ adherent spheroids were also cultured under hypoxic, low-glucose conditions to compare to their reoxygenated adherent spheroid counterpart. Real-time quantitative PCR was conducted using the QuantStudio 6 Pro (Thermo Fisher Scientific). Data normalization and ΔCT (Supplementary Table S2) value comparisons between experimental groups were carried out using Qiagen’s online analysis tools. A heatmap comparing ΔCT values across different stages of ovarian cancer dissemination was generated using Prism 10 (GraphPad). Data shown are the mean of 2-3 replicates.

### Western blot

Western blotting was performed to quantify the protein expression in adherent cells, spheroids, and adherent spheroids. Proteins were extracted with RIPA buffer supplemented with protease and phosphatase inhibitors (Thermo Fisher Scientific). Protein concentrations were determined using the Pierce BCA Protein Assay Kit (Thermo Fisher Scientific). Proteins were separated on an 8% Bioacrylamide SDS-PAGE gel and transferred onto PVDF membranes (Bio-Rad). Membranes were blocked with 5% bovine serum albumin (BSA) in 1× Tris-buffered saline containing 0.1% Tween-20 (TBST).

Primary antibodies were used to detect the integrin monomers ITGα2 (Thermo Fisher), ITGα3 (Thermo Fisher), ITGα4 (Millipore), ITGα5 (Cell Signaling), ITGαV (Millipore), ITGα6 (Thermo Fisher), ITGβ1 (Novus Biologicals), and ITGβ3 (Thermo Fisher). IRDye 680RD and IRDye 800CW secondary antibodies were used for visualizing the protein bands using the LI-COR Odyssey CLx imaging system (LI-COR Environmental). Protein expression levels were normalized to the housekeeping protein α-tubulin. Data are presented as mean ± SEM of at 3 to 4 biological replicates.

### Immunofluorescence staining of actively outgrowing cells

Immunofluorescent staining was performed as described (Roberts et al., 2005) in adherent cells and adherent spheroids growing on glass coverslips using primary antibodies directed against ITGα2 (Thermo Fisher), ITGα3 (Thermo Fisher), ITGα4 (Millipore), ITGα5 (Cell Signaling), ITGαV (Millipore), ITGα6 (Thermo Fisher), ITGβ1 (Novus Biologicals), and ITGβ3 (Thermo Fisher) and the appropriate secondary antibodies (Invitrogen). After mounting onto glass slides using ProLong™ Gold Antifade Reagent with DAPI (Invitrogen), images of adherent cells and cell outgrowth around adherent spheroids were acquired with a Nikon 80*i* epifluorescence microscope and NIS-Elements BR3.0 software. Negative controls are shown in Supplementary Figure S1. Images were analyzed with ImageJ and quantitated with Prism 10 (GraphPad). Data are presented as mean ± SEM from three independent experiments performed in triplicate with approximately 30 images of the cells.

### 3D reconstruction of spheroids via laser Scanning confocal microscopy and protein quantitation using Imaris

To perform 3D imaging of spheroids, optical clearing technique was performed with the Miltenyi MACS Clearing kit as per the manufacturer’s instructions. Briefly, fixed spheroids were transferred to a polypropylene Eppendorf tube and permeabilized with the MACS permeabilizing solution to allow for the penetration of the primary and secondary antibodies into the spheroids. Nuclei were stained with a DAPI solution (Thermo Fisher). After washing in 1X antibody staining solution, the spheroids were dehydrated in 50%, 70%, and 100% ethanol with 2%Tween 20, cleared in Miltenyi MACS clearing solution and transferred to 35 mm^3^ glass bottom confocal Petri dishes (Cellvis). The same procedure was used for adherent spheroids, but all the steps were performed in 33 mm^2^ glass bottom confocal Petri dishes. Optical Z stack slices were taken at 40X magnification with a Zeiss LSM880 laser scanning confocal microscope with Airy scan using Zen2 Blue software. Spatial localization of the proteins in the center slice of the non-adherent spheroids was determined by marking the outer rim of the mid-section with a circle of the same width for all spheroids and quantitate the fluorescence in both the outer rim and the center using ImageJ.

Following image acquisition of the adherent spheroids, further image analyses were performed using Imaris 9.8.2 software (Oxford Instruments). In the 3D spheroids, the compartmental fluorescence quantification of nuclear vs*.* cytoplasmic and cell membrane protein expression was performed. By masking the FITC signal using the DAPI-defined nuclear segmentation, only the cytoplasmic and cell membrane signals were considered in the cytosol compartment, while the nuclear signal was analyzed using DAPI overlapped FITC within the nuclear regions. This approach prevents misclassification of cytoplasmic protein as nuclear. Adherent spheroids were reconstructed by first defining key regions, such as the “site of attachment” and “bottom,” using manual contouring for precise boundary definition. To improve axial resolution and achieve more isotropic 3D data, deconvolution and background subtraction were applied. Segmentation was performed to accurately isolate individual cells from their surroundings. Then object-based measurements were conducted to generate scatter plots correlate to spatial metrics such as X, Y and Z-axis with mean fluorescence intensity values. Experiments were conducted in 3 independent trials with 3-5 spheroids and each of the images provided 20,000 IMARIS data points.

### Cytotoxicity alamarBlue assay

The cytotoxicity of the integrin inhibitors BT3033, E7820, ITG-IN-TFA, GLPG0187, TC I-15, AIIB2, Cyclo (RGDyK), and K34c was tested using the alamarBlue assays as described (Grieco et al., 2023b) in MOSE-L and MOSE-L_TIC*v*_ monolayers and spheroids. Drug properties are shown in the Supplementary Table S3. Determination of drug concentrations that do not overtly induce apoptosis and controls are shown in the Supplementary Figure S1. Spheroids were seeded in ULA 96-well plates and maintained for 24 h under HO LG conditions, while monolayers were plated in 96-well plates under NO HG for 24 h. Cells were then treated with drugs at the indicated concentrations in DMEM containing 2% FBS for 24 h and 48 h before AlamarBlue (10 µL/well) was added to wells. Fluorescence was measured with a Cytation 5 plate reader (540/590 nm). The highest growth-inhibitory but non-toxic concentrations were used for aggregation and outgrowth assays.

### Spheroid aggregation and adhesion imaging

For the spheroid aggregation assay, MOSE-L and MOSE-L_TICV_ cells were incubated with BT3033 (0.5, 5 µM), E7820 (15, 20 µM), ITG-IN-TFA (25, 30 µM), GLPG0187 (1.3, 5 nM), TC-I 15 (20, 25 µM), AIIB2 (20, 30 µM), Cyclo (RGDyK) (5, 15 µM), and K34c (10, 15 µM) in ultra-low adherence plates for 24 h under HO LG conditions. Aggregation was assessed qualitatively at 20X magnification using a Nikon Eclipse Ts2R microscope with NIS Elements BR5.43 imaging software. For adhesion assays, the same drug treatments were applied after spheroid formation. Images were taken following adhesion under HO LG conditions (24 h) and following reoxygenation under NO HG conditions (48 h).

### Statistical analysis

All statistical analyses were performed using GraphPad Prism 10. Data are presented as mean ± SEM, with biological replicates of at least *n* = 3. Comparisons between groups were analyzed using one-way or two-way ANOVA or paired t-test as indicated. Intensity measurements for both spheroid and adherent spheroid conditions were obtained using Imaris software which provided the means of 20,000 data points per trial. Spatial and intracellular differences were calculated with paired t-tests. Results were considered statistically significant at p < 0.05.

## Results

### Differential gene expression profile during ovarian cancer progression and metastatic dissemination

**FIGURE 1:**
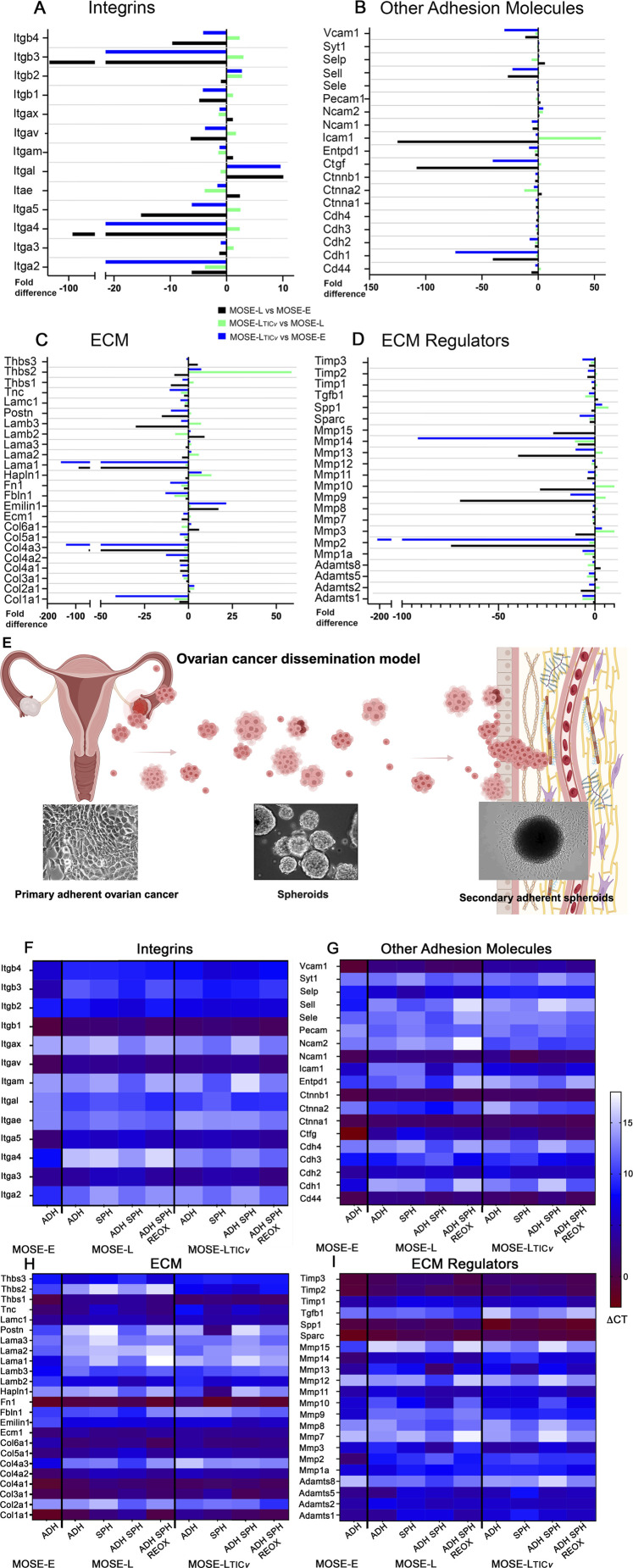
Differential gene expression during progression. Fold changes in gene expression patterns of **(A)** integrins and **(B)** other adhesion molecules, **(C)** extracellular matrix, and **(D)** cell adhesion and ECM-related regulatory genes during **(E)** MOSE progression from benign (MOSE-E) to slow- (MOSE-L) and fast-developing disease (MOSE-L_TIC*v*_) (generated with Biorender). Heatmap of changes in **(F)** integrin, **(G)** other adhesion molecules, **(H)** ECM and **(I)** their regulators expression in adherent monolayers (Adh), after aggregation (SPH), and adhesion in hypoxia (ADH SPH HOLG), and in adherent spheroids after reoxygenation (ADH SPH REOX).

Given the critical role of integrins and integrin regulators in mediating cell–cell and cell-ECM adhesion, we first assessed changes in integrin expression associated with ovarian cancer progression. We compared fold changes in gene expression levels between the benign MOSE-E, MOSE-L (representing slow-developing disease), and MOSE-L_TIC*v*_ (aggressive, fast-developing disease). In general, the expression levels of individual integrin subunits were very different between the cancer stages (Supplementary Table S2). As shown in Figure 1A, highly expressed in both cancer cell lines were *ITGα3*, *ITGα5*, *ITGαV*, and *ITGβ1*. No, or very low expression (CT > 35) was noted for *ITGαE*, *ITGαL*, *ITGαM*, and *ITGαX*. This was expected since these integrins are expressed mostly in immune cells (Dotzer et al., 2021). Most integrins were lower in MOSE-L compared to the benign MOSE-E (black bars) which was partially reversed in the highly aggressive MOSE-L_TIC*v*_ (green bars). Only *ITGα2* expression further decreased in the aggressive disease (Figure 1A). Other adhesion molecules and regulators such as *CD44*, *CTNNA1*, *CTNNB1, NCAM1* and *VCAM1* were highly expressed in the MOSE cells while others were either low (*CDH2*, *CTGF*, *ICAM1*) or were not expressed (Figure 1B; Supplementary Table S2). A similar expression pattern as observed in the integrin expression with a lower expression in the MOSE-L than MOSE-E but higher in MOSE-L_TIC*v*_ was observed for *CD44, CTGF* and *ICAM1* (Figure 1B). This elevation correlates well with their silencing in earlier stages of ovarian cancer but increased expression in later stages (*CTGF*) (Cannistra et al., 1995; Kikuchi et al., 2007), and contribution to an aggressive phenotype (*CTGF*, *NCAM*) (Cannistra et al., 1993; Zecchini et al., 2011), and stemness (*CD44*) (Zhang et al., 2008; Strobel et al., 1997).

Important for ovarian cancer is the expression of ECM proteins such as collagens, *FN1*, *TNC*, and their regulators (*MMPs*, *SPP1*, *TIMPs*). We saw similar changes in their expression during MOSE progression with a reduced expression in the MOSE-L compared to the benign cells, but a higher expression in the MOSE-L_TIC*v*_ (*Col2A1*, *LAMA2, LAMB3, THBS2, MMP3,9,10,13,* and *SPP1*). However, most of these genes were still highly expressed (Figures 1C,D; Supplementary Table S2) and we have shown previously that both COL1A1 and FN1 are rapidly secreted by MOSE spheroids (Shea et al., 2023). *MMP3* expression was higher in the MOSE-L_TIC*v*_ than in the benign MOSE-E (Figure 1D), which correlates well with studies in human tissue that show an elevation of *MMP3* in higher-grade ovarian cancer tissues (Wang et al., 2019). *SPP1* (osteopontin), a glycoprotein overexpressed in many cancer types and associated with a poor prognosis (Wei et al., 2020), was overexpressed in the MOSE-L_TIC*v*_ cells while the expression of *SPARC*, an inhibitor of ovarian cancer, growth and invasion (Said et al., 2007) was progressively reduced during MOSE progression albeit still highly expressed (Figure 1D). *LAMA1* and *LAMC1* were sequentially reduced in the cancer cells, while *LAMB2* was higher in the cancer cells. This correlates well with reports of their expression patterns in human ovarian tumor tissues (Kuhn et al., 2012). These data suggest that while the changes in the adhesion molecules, ECM components, and their remodeling enzymes were modest, together they may represent complex, stage-specific shifts that support cancer progression. Similar shifts higher expression of these adhesion-related molecules in MOSE-L_TIC*v*_ may be contributing to their increased metastatic potential and aggressive behavior.

### Gene expression changes during aggregation and adhesion

To mimic the transcoelomic metastasis of ovarian cancer rather than using cell monolayers we generated spheroids of the MOSE-L and the aggressive MOSE-L_TIC*v*_ cells in hypoxic and low glucose culture conditions as observed in ascites (Schielzeth et al., 2020). We then let them adhere in the same culture conditions (adherent spheroids) and then mimicked gaining access to oxygen and nutrients (reoxygenation-reox) by changing the culture conditions to normoxia and high glucose media (NOHG) (Figure 1E). Highly expressed in all culture conditions in both MOSE-L and MOSE-L_TIC*v*_ were *ITGβ1*, *ITGα3, ITGαV*, and *ITGα5*. This is in agreement with their expression in human tumors (Ahmed et al., 2002; Ahmed et al., 2005). As shown in Figure 1F, several integrins were higher expressed after aggregation (*ITGβ2* and *ITGβ3)* while only *ITGα3* was downregulated by aggregation in both cell lines. Other integrins changed in a cell type-specific manner. In MOSE-L_TIC*v*_, aggregation increased the expression of *ITGα2*, *ITGα4*, *ITGβ2*, and *ITGβ4,* suggesting a contribution to cell-cell adhesion and survival. *ITGα3*, *ITGα5*, and *ITGβ1* were reduced in MOSE-L_TIC*v*_ spheroids. In contrast, integrin expression either did not change in MOSE-L or in the opposite direction from MOSE-L_TIC*v*_. Adhesion of the spheroids in HO LG conditions had a limited effect on integrin expression in MOSE-L_TIC*v*_ where we observed an increased expression of *ITGβ1* and *ITGα5* while *ITGα2* and *ITGα4* expression levels were lower (Figure 1F). In MOSE-L, *ITGα2*, *ITGα4*, *ITGβ2*, and *ITGβ3* were increased while *ITGα5* was decreased after spheroid adhesion. In contrast, exposure of the adherent spheroids to reoxygenation caused an increase in most integrins in both cell lines. The fast-developing disease was characterized by more pronounced increases in all integrins but *ITGβ2*, *ITGβ3*, and *ITGβ4* which decreased after reoxygenation. These results imply differential roles of individual integrins in aggregation and adhesion and at different stages of the disease.

There were little changes in the other highly expressed adhesion molecules in MOSE-L after aggregation but an increased expression of *CD44*, *CDH2*, and *NCAM1* in MOSE-L_TIC*v*_ (Figure 1G). Adhesion of the spheroids caused an increase in the expression of *CTGF*, *ICAM1*, and *CTNNA1* in both cell lines while all other shifts were cell type specific. Notably, *NCAM1* was decreased in in MOSE-L_TIC*v*_ but still higher expressed than in MOSE-L which expressed higher levels of *VCAM1* after aggregation. In addition to adhesion, both proteins guide immune cell trafficking and recruitment. Reoxygenation increased adhesion molecules mostly in the MOSE-L_TIC*v*_ (*CD44*, *CDH1*, *CTNNA1*) while only *CDH3* and *CTGF* were upregulated and *ICAM-1* was downregulated in both cell lines (Figure 1G).

There was also very little overlap in ECM genes that responded to aggregation with an increase (*ECM1*) or a decrease in expression levels (*COL3A1*, *COL4A1*) between the slow- and the fast-developing disease. The aggressive MOSE-L_TIC*v*_ showed robust increases in several collagens, *FN1*, *HAPLN1, LAMB2*, *LAMC1*, *POSTN, TNC*, and *THBS2* after aggregation but reduced levels of *LAMB2* and *TNC* (Figure 1H), suggesting a role of these genes in cell-cell adhesion. After adhesion of the spheroids, several collagens (notably *COL1A1* and *COL3A1*) were upregulated in the MOSE-L_TIC*v*_ but reduced in the MOSE-L spheroids. While still highly expressed in all stages of dissemination, *FN1* was highly elevated after reoxygenation in both cell lines as were *COL1A1*, *COL4A2*, *COL5A1*, and *TNC*.

ECM regulators that were highly expressed in both cell lines (*TIMP2*, *TIMP3* and *SPP1*) were reduced after aggregation but higher in adherent spheroids (Figure 1I). In contrast, *SPARC* expression was reduced after aggregation with a gradual increase after aggregation and adhesion of MOSE-L_TIC*v*_ spheroids and reoxygenation. MMPs were mostly upregulated by aggregation and further by adhesion of the spheroids in MOSE-L. We observed little changes in these genes in MOSE-L_TIC*v*_ after aggregation and reduced expression after adhesion of the MOSE-L_TIC*v*_ spheroids. Reoxygenation of the adherent spheroids increased several of the highly expressed ECM regulators in both cell lines. These data suggest that the interactions of specific integrins, adhesion molecules, ECM and their regulators play a role at specific stages during ovarian cancer dissemination. Our data also show a distinct difference between the responses of the cell types to these culture conditions that may contribute to the aggressive phenotype of the MOSE-L_TIC*v*_.

### Differential integrin expression across stages of ovarian cancer progression and metastasis

**FIGURE 2:**
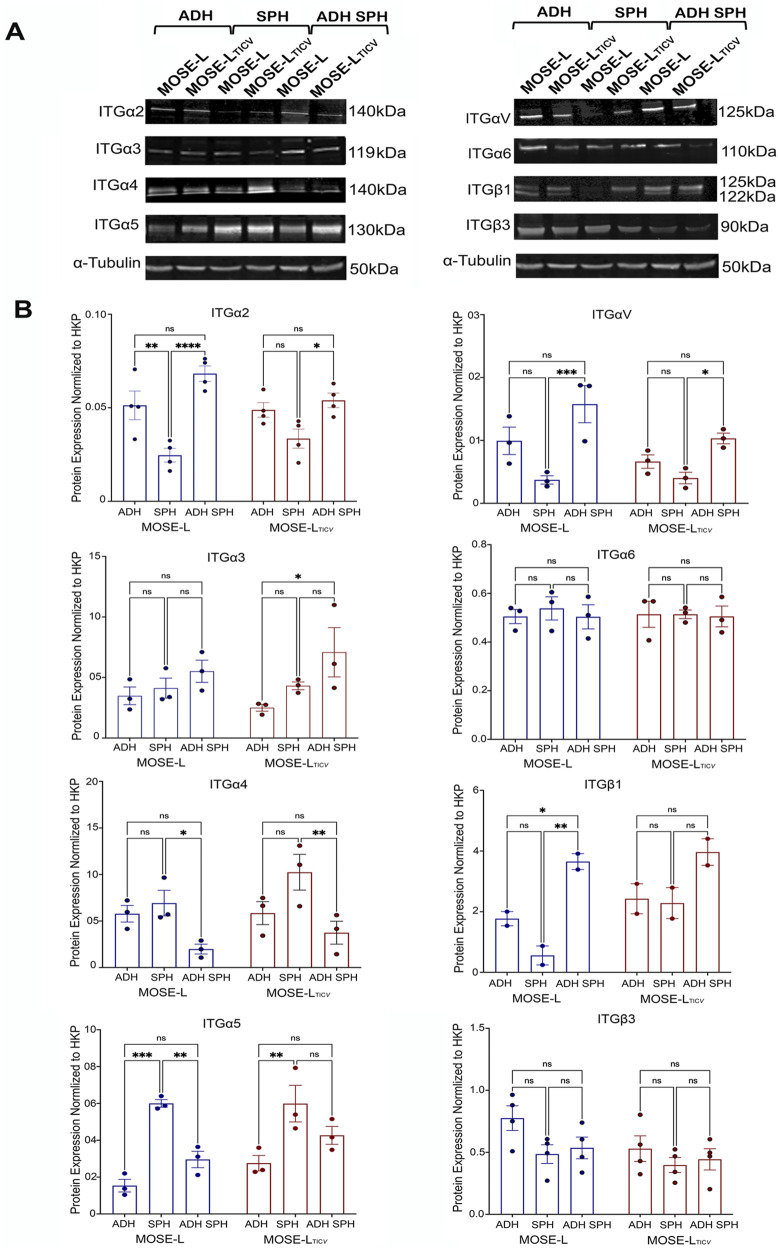
Differential protein expression during metastasis. **(A)** Comparison of adherent cells (ADH), spheroids (SPH), and adherent spheroids (ADH SPH) by Western blot analyses. **(B)** Quantitation of protein changes. *p < 0.05; **p < 0.01; ***p < 0.001 as determined by two-way ANOVA.

Integrins are recognized as key players in cancer, contributing significantly to processes such as migration, tumor invasion, and metastasis (Hamidi and Ivaska, 2018; Haake et al., 2024). Altered integrins expression is commonly linked to the aggressive nature of cancer cells by enhancing their capacity to invade nearby tissues and metastasize to distant locations (Hamidi and Ivaska, 2018). Thus, we focused next on integrins and compared changes in their protein expression during the metastatic spreading of ovarian cancer (Figure 1B) to the qPCR results above. Our results revealed distinct patterns of integrin expression at each stage: a gradual increase (ITGα3) or decrease (ITGβ3) in integrin expression as ovarian cancer progresses to advanced stages during dissemination, a decreased expression after spheroid formation but increased after spheroid adhesion (ITGα2, ITGαV, ITGß1), or an increased expression in spheroids but decreased after spheroid adhesion (ITGα4, ITGα5 and ITGα6) (Figures 2A,B). These results are similar to qPCR data above, confirming expression changes at both the gene and protein levels.

### Spatial and intracellular integrin expression in 3D spheroids

**FIGURE 3:**
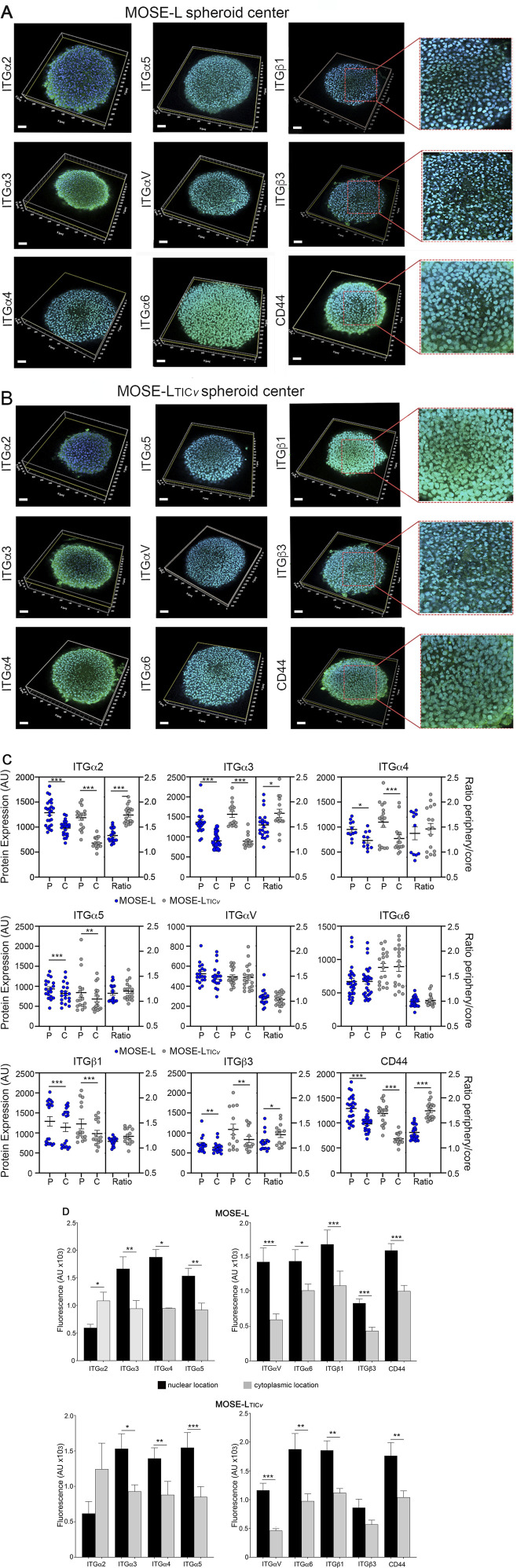
Spatial integrin expression in ovarian cancer spheroids. Integrin and CD44 expression in the center section of non-adherent **(A)** MOSE-L and **(B)** MOSE-L_TIC*v*_ spheroids grown in low glucose and hypoxia; Bar = 30 μm. **(C)** Mean fluorescence intensity in the periphery -P- or the core -C- of the spheroids. The ratio of periphery over core expression is shown on the right side for each protein. **(D)** Quantitation of nuclear versus cytosolic expression. *p < 0.05; **p < 0.01; ***p < 0.001 as determined by paired t-test.

Changes in the expression of integrins such as ITGβ1 or ITGα2 were less pronounced than expected after review of the current literature (Masi et al., 2023; Strobel and Cannistra, 1999; Dotzer et al., 2021). We hypothesized that integrin expression changes may occur only at specific sites of the spheroids and may be masked by the unresponsive bulk of the spheroid when quantitated by qPCR or Western blotting Therefore, we next investigated spatial differences in integrin expression in the spheroids, selecting integrins that showed a change in expression after aggregation and adhesion. Here, we employed a 3D reconstruction model of laser-scanning confocal microscopy images. Shown in Figure 3 are distinct differences in the distribution of highly expressed integrins and CD44 in the center section of non-adherent MOSE-L (Figure 3A) and MOSE-L_TIC*v*_ (Figure 3B) spheroids. Most integrins were significantly higher expressed in the periphery of the spheroids as demonstrated by a ratio of protein expression in the periphery over expression in the core >1 while ITGαV and ITGα6 were uniformly expressed throughout the spheroids (ratio periphery/core ∼1) (Figure 3C). The ratio of ITGα2, ITGα3, and ITGβ3 expression in the periphery versus core was significantly higher in the MOSE-L_TIC*v*_ than in the MOSE-L while there was no difference observed for the other integrins. Interestingly, in addition to a different spatial expression of the integrins in the spheroids, we also observed differences in the intracellular location of the integrins. Therefore, we measured the mean fluorescence intensity of integrin expression in the nucleus or the cytoplasm and plasma membrane with the Imaris software as described in the method section. In both cell lines, only ITGα2 was expressed mostly in the cytosol and plasma membrane (not significant for MOSE-L_TIC*v*_) while all other integrins were significantly higher expressed in the nucleus (Figure 3D). In general, cells in the periphery had a higher expression of integrins in the cytosol, while cells localized at the core of the spheroid showed a predominant nuclear location (see Figures 3A,B, right column). While investigations into the function of nuclear integrins were beyond the scope of this investigation, the potential impact of the integrins on gene expression and, thereby, on the phenotype of the cancer cells is of great importance. Similarly, the key adhesion and signaling molecule CD44 was higher expressed in peripheral regions of the spheroids with an intracellular location shift from nuclear expression in the spheroid core to membrane/cytosolic in the peripheral cells (see z-stack in Supplementary Video S1).

### Site-specific integrin expression after spheroid adhesion

**FIGURE 4:**
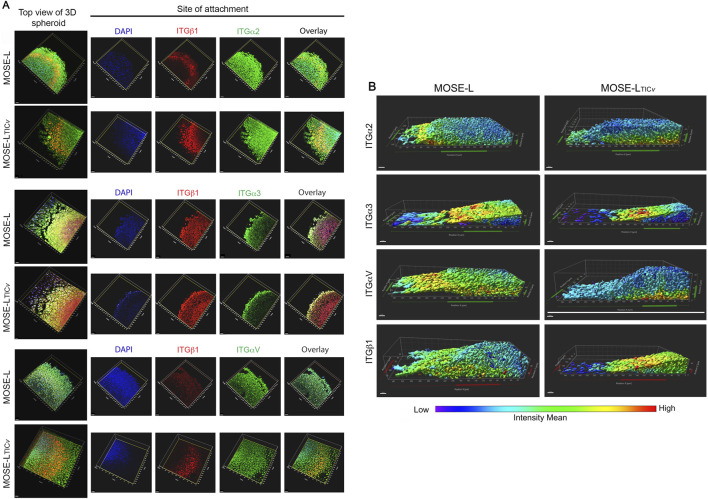
3D spatial integrin expression in adherent spheroids. Spheroids were allowed to attach to tissue culture-treated plates in low glucose and oxygen conditions. Proteins were visualized via immunofluorescent staining and confocal imaging. **(A)** Integrin expression at the site of adhesion. Left column: top view of adherent spheroid. **(B)** Cross section of adherent spheroids cut at top of spheroid and image reconstruction of the z-stack. Height: 40–60 μm dependent on the flattening of the spheroid after attachment. Bar = 20 μm. Blue represents low to no expression while green and yellow and indicate higher expression and red highest expression.

We then determined the impact of adhesion on integrin expression and distribution using laser scanning microscopy and 3D reconstruction of the Z-stack images. We focused specifically on ITGα2, ITGα3, ITGαV, and ITGβ1 expression and localization since these integrins were upregulated in adherent spheroids as determined by Western blot (Figure 2) albeit this provided only a bulk expression profile without spatial distribution analysis. The Imaris software allowed us to visualize integrin distribution and color mapping the mean intensity levels of integrin expression at the adhesion site (Figure 4A) or throughout the adherent spheroid (Figure 4B). As shown in Figure 4A, ITGα2 and ITGαV showed a strong expression at the site of adherence and in the outgrowth in both MOSE-L and MOSE-L_TIC*V*_ models while ITGα2 and ITGβ1 were found highly expressed in the cells growing out from the spheroid after attachment. ITGβ1 expression was also found on the site of adherence but that was more variable between spheroids. The 3D reconstruction of the attached and somewhat flattened spheroids in Figure 4B (cut off vertically at the middle of the attached spheroid with outgrowth areas on the left side) shows that adhesion differentially affected integrin expression. While there was a low ITGα2 expression in the outer layer of the floating spheroids and very little expression in their core (see Figures 3A,B), after adhesion ITGα2 expression was apparent at the site of adhesion. Importantly, this was not restricted to a few cell layers but an intermediate intensity extended up to 40–50 μm into the spheroid core from the adhesion site. This change in expression was similar to the adhesion effects on ITGαV expression. In contrast, ITGα3 was mostly expressed at the site of spheroid outgrowth and at the spheroid surface while ITGβ1 was expressed at the adhesion sites but was highest in the outgrowing cells (Figure 4B). These results indicate that integrin expression during spheroid formation and attachment is not uniform, but rather spatially regulated, likely depending on each integrin’s role in anchoring, invasion, and interaction with the surrounding tumor microenvironment.

### Integrin expression in the cell outgrowth after spheroid attachment

**FIGURE 5:**
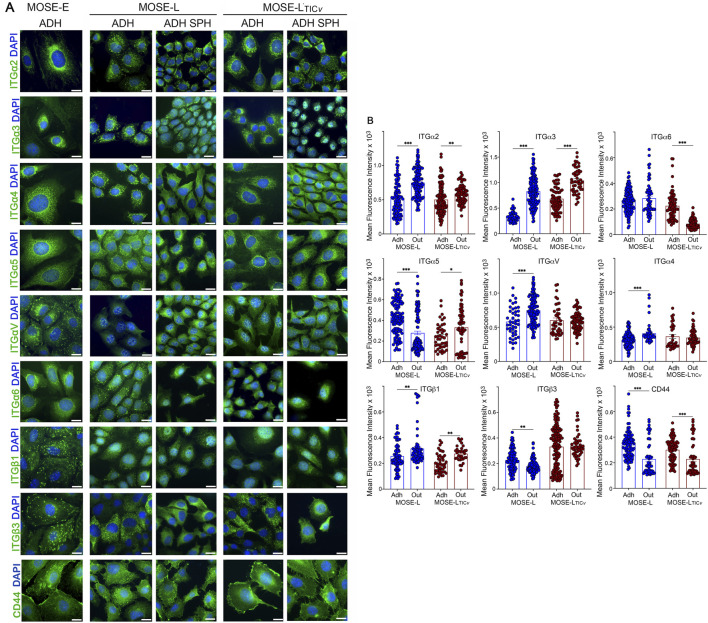
Plasticity of integrin changes. The integrin expression in the cells growing out from the adherent spheroids (ADH SPH) were compared to **(A)** the original cell monolayer (ADH) and **(B)** quantitated by ImageJ *p < 0.05; **p < 0.01; ***p < 0.001 as determined by paired t-test. Bar = 20 μm.

To investigate the plasticity of integrin expression, specifically their response to adhesion and proliferation, we compared the integrin expression in cells growing out from the adherent spheroids (outgrowth) to the original adherent monolayer cells to determine if the expression levels returned to the original levels. The expression levels of ITGα2, ITGα3, and ITGβ1 were higher in the outgrowth while only CD44 was lower in the outgrowth in both cell types (Figures 5A,B). Other integrins were either higher (ITGαV), lower (ITGα5, ITGβ3) in the outgrowth of MOSE-L or were not different (ITGαα4). In contrast, in MOSE-L_TIC*v*_ we observed a significant increase in ITGα5 in the outgrowing cells and a reduced expression of ITGα4 while the expression levels of ITGαV, ITGα6, and ITGβ3 was not different from the original monolayers. Interestingly, the intracellular localization did not change for most integrins. Most integrins were expressed both in the cytosol and in the nuclei while ITGα2I and ITGβ3 were mostly expressed in the cytosol. Only ITGα3 and ITGβ3 expression changed from mostly cytosolic to mostly nuclear in MOSE-L_TIC*v*_ outgrowth (Figure 5A). In MOSE-E cells, integrins were expressed in the cytosol or at the focal adhesions but not in the nucleus. This focal adhesion localization was not observed in the cancer cells in either the monolayers or the outgrowths. This correlates well with the low focal adhesion number and size in the cancer cells we have observed before (Creekmore et al., 2013). These results suggest a role of the nuclear localization of integrins in the transformation and progression of ovarian cells and point to both an adaptation to culture conditions and the return to the expression patterns of the original monolayer for some integrins but a differential role of specific integrins in the outgrowing cells from the attached spheroids in a cell type-specific manner.

### Identification of integrins as targets to prevent aggregation and adhesion

**FIGURE 6:**
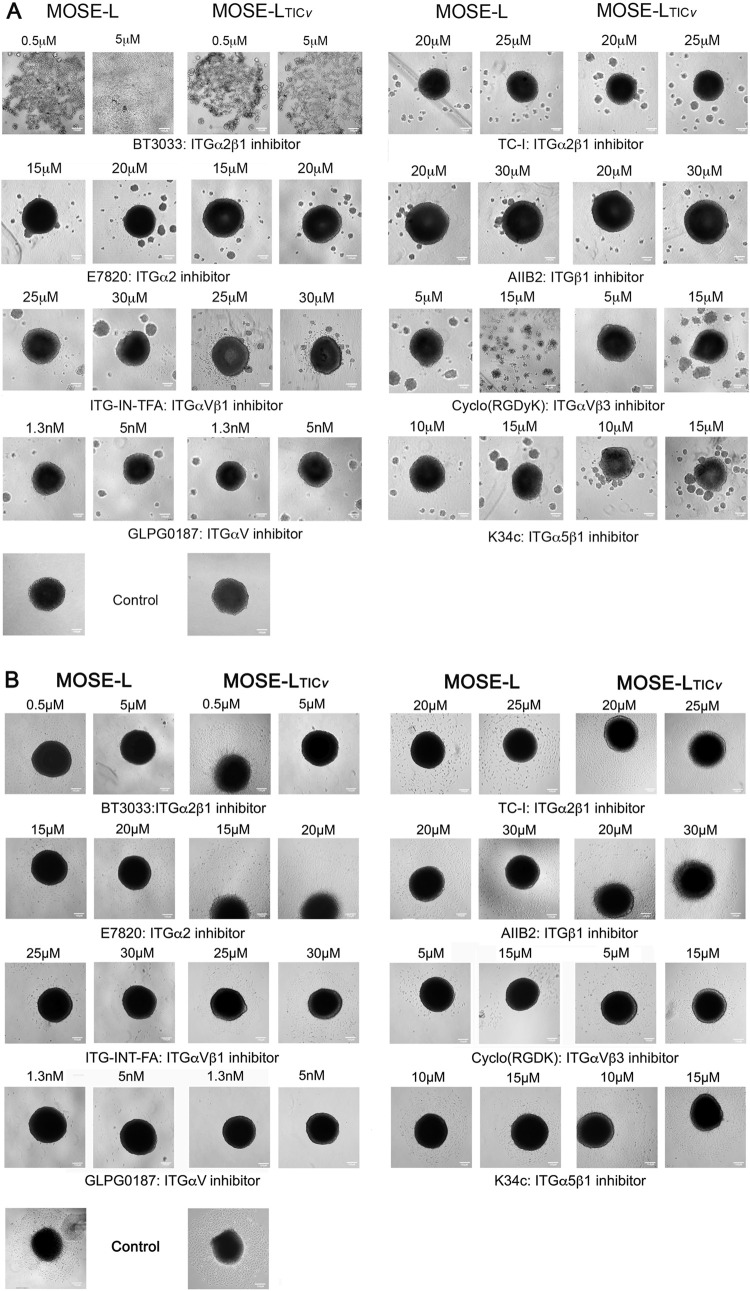
Integrin inhibitors differentially affect aggregation and adhesion. Cells were treated with the indicated inhibitors at **(A)** seeding to monitor impact on aggregation in low oxygen and glucose conditions or **(B)** after aggregation in low oxygen and glucose conditions (24 h) and transfer to high oxygen and glucose conditions for an additional 24 h (48 h) to determine the impact of inhibitors on adhesion. Bar = 100 μm.

To determine which integrin expression is critical for aggregation and adhesion, we used commercially available inhibitors against differentially expressed integrins in concentrations that stunted growth but did not affect cell viability (Supplementary Figure S1) and monitored MOSE aggregation and adhesion. As shown in Figure 6A, the MOSE cells form a single, tight spheroid after 24 h incubation in low oxygen and glucose conditions. Inhibition of ITGα2β1 with BT3033 prevented aggregation in both cell types in concentrations from 0.5 to 5 μM (Figure 6A and data not shown) while multiple small spheroids still formed in MOSE-L_TIC*v*_. However, neither TC-I, also an ITGα2β1 inhibitor, nor inhibition of the ITGα2 (with E7820) or ITGβ1 (with AllB2) monomers suppressed aggregation. Similarly, inhibition of ITGα5β1, ITGαVβ3, or ITGαV did not prevent aggregation. Only CycloY (RGDyK), a specific ITGαVβ3 inhibitor, suppressed aggregation in MOSE-L at 15 μM while the fusion to a single spheroid but not aggregation *per se* was observed in MOSE-L_TIC*v*_. The multiple spheroid formation was also apparent in the treatment with the other integrin inhibitors (Figure 6A). These results suggest that either only the ITGα2β1 heterodimer is critical for aggregation, or the inhibitors do not effectively suppress integrin signaling.

We next investigated the effect of these inhibitors on adhesion and outgrowth. Inhibition of ITGα2β1 with BT3033 at 0.5 μM had no effect in either MOSE line while 5 μM (the dose that prevented aggregation) inhibited adhesion and, subsequently, outgrowth and viability of the MOSE spheroids (Figures 6B,C). In contrast, neither TC-I nor E7820 nor AII2B inhibited adhesion and had little impact on viability. However, while the outgrowth of MOSE-L_TIC*v*_ spheroids was not inhibited, the cells growing from MOSE-L spheroids had a migratory phenotype rather than the outgrowth as a sheet as observed in the controls. Inhibition of ITGαVβ1 with ITG-INT-FA or ITGαVβ3 with (Cyclo (RGDK)) did not suppress adhesion but reduced drastically the outgrowth in MOSE-L and decreased the viability with less effect on MOSE-L_TIC*v*_ (Figures 6B,C). Inhibition of ITGαV using GLPG0187 completely prevented adhesion and outgrowth in both MOSE-L and MOSE-L_TIC*v*_. Inhibition of ITGα5β1 did not prevent adhesion but spheroid outgrowth and viability were reduced in MOSE-L. Thus, ITGαV may play a dominant role in cancer cell adhesion and outgrowth while inhibition of other integrins may reduce the activation of signaling pathways that induce proliferation, often in a cell-type specific manner. This is currently being investigated in our lab.

## Discussion

Ovarian metastasis is a dynamic process that requires expression and functional changes to enable the adaptation to the microenvironment transforming from adhesion to exfoliation and aggregation, to adhesion of the spheroids and, finally, outgrowth and invasion at distant sites. Integrins are involved in every stage of this metastatic progression. Here we assessed changes in integrins and associated adhesion molecules during ovarian metastasis in culture conditions that mimic the change of microenvironment the metastases encounter in the peritoneal cavity. Our studies revealed a pattern of highly expressed integrins (*ITGα3*, *ITGα5*, *ITGαV* and *ITGβ1*) with either little changes during progression or a modestly lower expression in the cancer cells with the most aggressive disease having higher levels of these integrins than the MOSE-L. Ligands for ITGβ1-containing heterodimers are FN1, several collagens and laminins, SPP1, thrombospondin and others while the α subunits determine substrate selectivity (Humphries et al., 2006). Our results show that in parallel to the integrin expression, integrin ligands FN1, several collagens, and laminins and SPP1 were also highly expressed in the MOSE cells with higher levels in the MOSE-L_TIC*v*_. FN1 is abundant in malignant ascites (Fritz et al., 2020) and interacts with ITGα5β1 to promote spheroid formation and adhesion (Burleson et al., 2004a; Casey et al., 2001). Collagens are abundant in tumor stroma and facilitate the clustering of integrins to promote cell survival, adhesion, migration and invasion (Gilkes et al., 2014). The omentum, the first metastatic site for ovarian cancer cells, is also enriched in collagens and ECM remodeling enzymes such as MMP2 and MMP9 (Huang et al., 2020). In ovarian tumor samples, ECM proteins such as collagens, laminins, tenascin C, and ECM regulators such as MMPs and TIMPs (Salani et al., 2007; E et al., 2021; Kim et al., 2006) were highly expressed and were linked with advanced stages of the disease and reflective of aggressive tumor behavior. Thus, while the expression changes of integrins were modest, they were accompanied by changes in the expression of other adhesion-and ECM proteins and their regulators and, thus, likely reflect a broader reprogramming of adhesion networks that may facilitate the adaptations necessary at different steps of ovarian cancer progression and dissemination.

Both MOSE lines readily aggregate when cultured in ultra-low adherence plates to single tight spheroids. While we saw a differential expression of *ITGα4*, *ITGβ2*, and *ITGβ3* between the cell lines it is unclear if these changes are critical for aggregation or if the highly expressed integrins *ITGα3*, *ITGα5*, *ITGαV* and *ITGβ1* that have been shown to promote spheroid formation (Sodek et al., 2009; Burleson et al., 2004a) are more important. It is also feasible that changes in lower expressed integrins are altering both the composition of integrin dimers and the balance of α and β subunits to promote aggregation and activation of survival signals. This needs to be investigated in more detail when targeting aggregation to reduce the survival of ovarian metastases. We also observed moderate changes in integrins, ECM and regulators after adhesion but increased levels of *ITGα3*, *ITGα5*, *ITGαV*, and *ITGβ1* after reoxygenation of the adherent spheroids, mimicking gained access to oxygen and nutrients. These integrins have been shown to promote adhesion, spheroid disaggregation and spreading, proliferation, invasion and migration (Shield et al., 2007; Casey et al., 2001; Yin et al., 2025; Moffitt et al., 2019). The increased expression of FN1 and Col1 also promotes spheroid disaggregation and spreading (Burleson et al., 2004a; Burleson et al., 2004b; Burleson et al., 2006) while the increased levels of ECM remodeling enzymes (several MMPs, SPP1, TIMPs mostly in MOSE-L_TIC*v*_) support invasion (Carey et al., 2021). Thus, the combined increase in adhesion molecules, ECM and ECM remodeling enzymes can support the adhesion and invasion of secondary sites.

The changes in integrins, ECM and their regulators after adhesion suggest that adhesion activates signals that alter the more dormant phenotype of the spheroids. Indeed, we observed increased integrin expression at the adhesion site (ITGα2, ITGαV) or at the spheroid surface and sites of spheroid outgrowth (ITGα3, ITGβ1). This site-specific expression change may be partially masked in gene or protein analyses due to the unresponsive bulk of the spheroids but could affect the formation of integrin heterodimers with the highly expressed integrins and provide strong adhesion-mediated signals to promote adhesion and outgrowth. The spatial distribution in the non-adherent spheroids (peripheral versus core) did not predict integrin expression after adhesion. The spatial heterogeneity of integrin expression between spheroid periphery and core but higher expression at adhesion or proliferation sites may reflect functional compartmentalization and adaptation to microenvironmental cues and receptor-activated signaling to switch from a more dormant state to active proliferation. Thus, integrin expression during spheroid formation and attachment is not uniform but spatially regulated, likely reflecting each integrin’s role in anchoring, proliferation, motility and invasion, and interactions with the tumor microenvironment.

Interestingly, several integrins and CD44 showed a robust nuclear accumulation over membrane expression. While cytoplasmic integrins may be the result of their trafficking between the cell membrane and cytoplasm as well as possible recycling processes (Bouvard et al., 2013), the nuclear accumulation points to a novel role in gene expression regulation. While our results were observational in nature and not orthogonally validated, a nuclear translocation has been described for other classical membrane-bound receptors such as the EGFR family of receptor tyrosine kinases or GPCRs. This translocation was linked to alternative intracellular roles such as modulating transcriptional activity or participating in nuclear signaling cascades (Mills, 2012; Gold et al., 2013). However, there is very little information on integrins translocating to the nucleus and their role in cancer development and metastasis. ITGαVβ3 has been identified in the nucleus of patient-derived tissues and high-grade ovarian cancer cells where increased expression of genes and interactions with nuclear proteins contributed to enhanced proliferation, migration, and oncogenic signaling (Seraya-Bareket et al., 2020). Similarly, ITGαV but not ITGβ3 has been shown to be a co-activator of *COX2*, *ERa, HIF1a* and *TRb1* transcription (Lin et al., 2013). In contrast, induced nuclear localization of ITGβ4 reduced cell viability in several cancer cell lines and their *in vivo* tumor formation (Liu et al., 2016). The prominent nuclear localization of several highly expressed integrins in the rapid outgrowth from the attached spheroids suggest a more nuanced role of integrins in activating adhesion-induced signaling pathways to promote cell proliferation and motility and directly regulating gene expression that support metastatic capacities. How the cellular responses differ from active nuclear versus membrane-bound integrins is unknown but needs to be investigated in more detail since these integrins may be important targets for suppression of metastatic outgrowth.

The specificity of integrin expression at distinct stages of metastatic dissemination suggests that inhibitors directed against these integrins could suppress metastasis by inhibiting aggregation or reduce adhesion and prevent secondary outgrowth. However, most commercially available inhibitors produced inconclusive results. Inhibition of ITGα2β1 with BT3033 but not inhibition of the individual monomers suppressed aggregation with no effect of either inhibitor on adhesion. Only the ITGαVβ1 inhibitor GLPG0187 inhibited adhesion of the spheroids while ITG-INT-FA (ITGαVβ1 inhibitor) reduced spheroid outgrowth similar to the ITGαVβ3 inhibitor Cyclo (RGDK) in both cell lines, suggesting a role of ITGαV in cell-cell and cell-ECM adhesion. Differences in their binding affinity and conformational changes of the target protein after binding of the inhibitors may explain why some drug candidates are effective while others targeting the same proteins are not. Alternatively, the involvement of multiple integrins and other receptors in aggregation and adhesion in a cell type-specific manner may render the inhibition of one target ineffective, or off-targets of the inhibitors that can reduce aggregation and adhesion could be involved. It is also possible that the intracellular localization of the integrins affect the cells’ response to the inhibitors. As nuclear integrins act as co-activators for transcription (see above), their function may be as prominent in gene expression regulation as in adhesion and adhesion-mediated signaling pathway activation, leading to activation of signaling pathways that induce drug resistance, increased survival and more. If intracellular or nuclear integrins do not or differently respond to the inhibitors is unknown. If the inhibitors target only membrane-bound integrins it is feasible that they do not impact the activity of the nuclear integrin pool. These limitations may have contributed to the lack of suppression of tumor formation and unchanged survival in clinical trials with inhibitors against integrin monomers or dimers in cancer and endothelial cells (Kobayashi et al., 2017). Thus, the development of inhibitors that target integrins at specific stages of the disease and nuclear-localized adhesion receptors may offer novel therapeutic avenues.

Integrin expression has been studied in patient-derived ovarian cancer tissues and ovarian cancer cell lines, and several adhesion molecules have been identified to be relevant in ovarian cancer. However, these reports were performed in tumor tissue with several cells in the tumor microenvironment expressing integrins, including fibroblasts, endothelial cells, platelets, and stromal cells (Desgrosellier and Cheresh, 2010) or in cell culture conditions that may not reflect physiological conditions such as monolayers with full access to oxygen and nutrients. In addition to the shown changes due to aggregation and adhesion, hypoxia that has been shown to selectively alter the expression of integrins that bind to collagen (ITGα1, ITGα11, ITGβ1), fibronectin (ITGα5, ITGβ1) or hemoglobin (novel ITGαDβ1) via HIF-1 and HIF-2 signaling (Ju et al., 2017; Koivunen et al., 2025; Velica et al., 2021). Further, the low nutrient levels in the peritoneal cavity also affect integrin expression (Nam et al., 2024). Thus, it is important to select culture conditions that are more relevant to the disease stage.

For our studies we were using our well-described ovarian cancer progression model that with MOSE-E, MOSE-L and MOSE-L_TIC*v*_ represent benign cells, slow and fast developing disease, respectively, allows us to model ovarian cancer dissemination in biologically relevant conditions. The transition from a normoxic, high-glucose environment at the primary site to a hypoxic, low-glucose state within the peritoneal cavity where spheroids are formed and attach to the mesothelium, can be mimicked in this model. This model also allows us to compare different disease stages from the same origin, the benign MOSE-E cells and avoid inter-individual difference in human cell lines originating from different women. While this is s murine model, we have shown that the phenotype reflects the human disease (Roberts et al., 2005; Creekmore et al., 2011). In future studies we can confirm our results by injecting single cells and spheroids into immunocompetent C57BL/6 mice and develop treatment strategies with specific inhibitors or inhibitor combinations.

**FIGURE 7:**
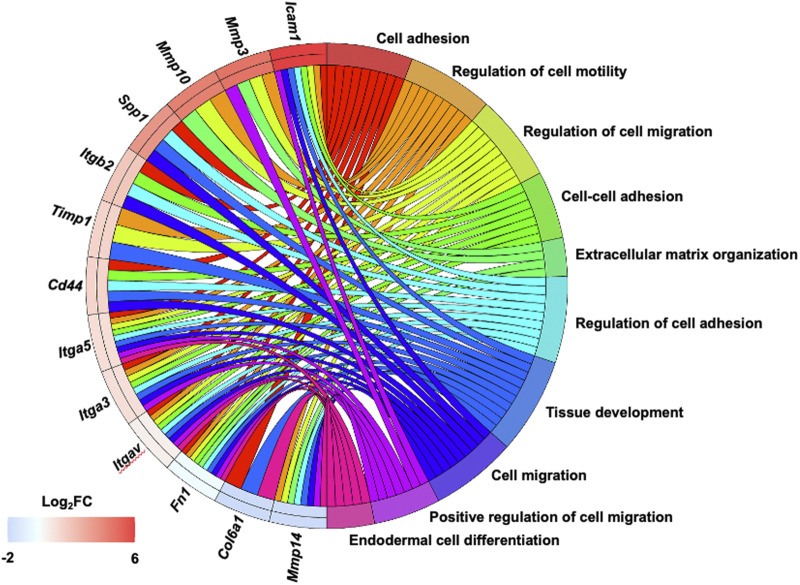
Gene Ontology chord plot of differentially expressed genes in MOSE-L_TIC*v*_ and their relationship to cellular functions. Gene expression levels in MOSE-L_TIC*v*_ adherent spheroids after reoxygenation were compared to those in MOSE-L and linked to cellular functions that may determine the aggressive phenotype of the MOSE-LTICv cells. Impact of fold changes (FC) gene expression changes on cellular functions listed in the top 10 gene ontology terms.

It is feasible that differences between the MOSE-L and the MOSE-L_TIC*v*_ in the expression of integrins, other receptors, and ECM and their regulators as well as the different response to several integrin inhibitors play a role in the aggressive nature of the MOSE-L_TIC*v*_. In general, the responses to changing culture conditions were more robust in the MOSE-L_TIC*v*_ with higher expression of several integrins, ECM and ECM regulators such as SPP1 and several MMPs that can contribute to the survival of the cancer cells after aggregation, and support ECM remodeling and proliferation and invasion after adhesion, especially after reoxygenation. Significant gene changes (fold change ≥1.5) between adherent MOSE-L and MOSE-L_TIC*v*_ spheroids after reoxygenation in the top 10 gene ontology terms shows that this overexpression strongly supports multiple processes such as adhesion, motility and migration and tissue development (ITGβ1 were not listed as related to the top 10 gene ontology terms) (Figure 7) that can contribute to an increased survival at metastatic sites and rapid outgrowth MOSE-L_TIC*v*_.

While not the focus of our investigation, CD44 is another critical surface receptor involved in ovarian cancer. The association of CD44 expression and the disease-free or overall survival is still unclear with several studies reporting a beneficial effect while others link CD44 expression to a worse prognosis (Sacks and Barbolina, 2015). In our model CD44 was lower expressed in the cancer cells but still at very high levels with little changes to culture conditions. In spheroids, CD44 is mostly expressed in peripheral cells and, similar to integrins, with a high expression in the nucleus. Nuclear translocation of full-length or CD44 fragments after ligation has been shown to contribute to transcriptional activation of genes that promote proliferation (Lee et al., 2009), glycolysis (Nam et al., 2016), progression, or reduced overall survival (Su et al., 2019) in addition to an increased transcription of CD44 itself (Okamoto et al., 2001). CD44 ligands are mainly hyaluronic acid and SPP1 which is highly expressed in the MOSE cells, but also fibronectin, and several collagens. Binding of SPP1 to CD44 has been shown to activate its association with Src and redistribution into lipid rafts with a concomitant enrichment and activation of ITGβ1 that promotes adhesion (Lee et al., 2008). SPP1 is also a ligand for ITG⍺Vb3 and has several binding sites for other integrins such as ITGαVβ1, ITGαVβ5 and ITGα51 (Shojaei et al., 2012). Thus, aggregation and adhesion signals may be generated by an interplay of the generation of specific integrin heterodimers, other adhesion receptors, a high expression of activating ligands and extracellular matrix regulators.

In summary, the dynamic interplay of receptors, their ligands and regulators allow for the adaptation of the cellular phenotype to exfoliation, aggregation and adhesion and promote the survival of disseminating ovarian cancer cells and a successful secondary outgrowth. These insights have significant translational implications. By identifying integrins critical to aggregation and survival during peritoneal dissemination and secondary site adhesion, therapeutic strategies can be developed to block these receptors to reduce peritoneal metastasis. This will ultimately improve 5-year survival rates. We envision that combinatorial treatments, targeting multiple signaling pathways, can be developed that will generate personalized treatments for patients, enhancing the effectiveness of therapies and enhancing the survival of women with advanced ovarian cancer.

## Data Availability

The raw data supporting the conclusions of this article will be made available by the authors, without undue reservation.
