# Supplementary material for: Dynamic integrin expression, atypical nuclear localization, and spatial distribution during ovarian cancer progression and metastasis
Source: Front Cell Dev Biol. 2026 Feb 25;14:1744403. doi: 10.3389/fcell.2026.1744403 (PMC12975958; doi:10.3389/fcell.2026.1744403)
Supplement: Supplementary file 1 [file Table1.docx]

| Cell Adhesion Molecules (CAMs) | |
| --- | --- |
| Cell-Matrix Adhesion | Itga2, Itga3, Itga4, Itga5, Itgad, Itgae, Itgal, Itgam, Itgav, Itgb1, Itgb2, Itgb3, Itgb4, Ctgf, Spp1 |
| cell to cell adhesion | Cdh1, Icam1, Vcam1, Pcam1 |
| Other Adhesion Molecules | Lama1, Lama2, Lama3, Lamb2, Lamb3, Lamc1, Postn, Tgfbi, Thbs1, Thbs2, Catna1, Ctnna2 (Catna2), Cntn1, Ctnnb1 (Catnb), Col5a1, Col6a1, Col8a1, Vcan, Emiln1, Fn1, Hapln1, Vtn |
| Extracellular matrix (ECM) Proteins | |
| ECM Structural Constituents | Col1a1, Col2a1, Col3a1, Col4a1, Col4a2, Col4a3, Col5a1, Col6a1, Col8a1, Hapln1, Lama1 |
| Basement Membrane Constituents: | Col4a1, Col4a2, Col4a3, Entpd1, Itgb4, Lama1, Lama2, Lama3, Lamb2, Lamb3, Lamc1, Sparc, Timp1, Timp2, Timp3 |
| ECM Proteases: | Adamts1, Adamts2, Adamts5, Adamts8, Mmp10, Mmp11, Mmp12, Mmp13, Mmp14, Mmp15, Mmp16, Mmp1a, Mmp2, Mmp3, Mmp7, Mmp8, Mmp9 |
| ECM Protease Inhibitors | Col4a3, Timp1, Timp2, Timp3 |
| Other ECM Molecules | Vcan, Ctgf, Ecm, Emilin1, Fbln1, Fn1, Postn, Spock1, Spp1, Tgfbi, Thbs1, Tnc |
| Housekeeping gene | Actn, B2m, Gapdh, Gusb, Hsp90ab1 |
|  | |

**Table S1.** Functional grouping of extracellular matrix and cell adhesion molecules
