# Supplementary material for: Dynamic integrin expression, atypical nuclear localization, and spatial distribution during ovarian cancer progression and metastasis
Source: Front Cell Dev Biol. 2026 Feb 25;14:1744403. doi: 10.3389/fcell.2026.1744403 (PMC12975958; doi:10.3389/fcell.2026.1744403)
Supplement: Supplementary file 2 [file Table2.docx]

| **Symbol** | **MOSE-E** | **MOSE-L ADH** | **MOSE-L SPH** | **MOSE-L**  **ADH-SPH Non-Reox** | **MOSE-L ADH-SPH**  **Reox** | **MOSE-L_TICV_**  **ADH** | **MOSE-L_TICV_**  **SPH** | **MOSE- L_TICV_**  **ADH SPH**  **Non-Reox** | **MOSE- L_TICV_**  **ADH SPH**  **Reox** |
| --- | --- | --- | --- | --- | --- | --- | --- | --- | --- |
|  | **ΔCT** | **ΔCT** | **ΔCT** | **ΔCT** | **ΔCT** | **ΔCT** | **ΔCT** | **ΔCT** | **ΔCT** |
| **Adamts1** | 3.62 | 3.64 | 6.97 | 6.95 | 4.33 | 6.32 | 8.66 | 6.24 | 5.47 |
| **Adamts2** | 4.39 | 7.26 | 6.26 | 4.90 | 5.53 | 5.99 | 5.42 | 5.01 | 4.99 |
| **Adamts5** | 3.27 | 2.89 | 6.47 | 5.76 | 4.38 | 4.92 | 8.23 | 4.75 | 5.28 |
| **Adamts8** | 15.28 | 12.13 | 12.50 | 10.34 | 12.59 | 14.01 | 13.77 | 16.28 | 13.17 |
| **Cd44** | 1.50 | 4.09 | 3.95 | 3.02 | 3.60 | 2.88 | 2.09 | 3.61 | 2.03 |
| **Cdh1** | 7.62 | 15.41 | 15.10 | 11.84 | 16.63 | 13.98 | 12.57 | 16.63 | 12.07 |
| **Cdh2** | 4.94 | 6.46 | 6.28 | 6.84 | 5.78 | 7.86 | 6.12 | 5.99 | 6.09 |
| **Cdh3** | 9.24 | 9.37 | 11.27 | 12.55 | 8.56 | 10.35 | 11.09 | 8.48 | 7.69 |
| **Cdh4** | 13.69 | 14.44 | 15.44 | 11.67 | 15.40 | 14.01 | 12.57 | 15.88 | 13.24 |
| **Cntn1** | 14.88 | 13.12 | 14.80 | 11.90 | 14.67 | 14.75 | 15.21 | 15.26 | 11.92 |
| **Col1a1** | -1.85 | 0.52 | 2.49 | 3.89 | 0.03 | 3.52 | 2.60 | 1.41 | 0.73 |
| **Col2a1** | 13.99 | 13.95 | 15.61 | 11.67 | 13.84 | 11.97 | 12.82 | 12.51 | 9.70 |
| **Col3a1** | 0.53 | 0.87 | 1.87 | 3.21 | 1.70 | 2.32 | 4.31 | 0.75 | 1.81 |
| **Col4a1** | -1.06 | 1.18 | 2.63 | 2.04 | 1.40 | 1.18 | 1.57 | 1.89 | 1.08 |
| **Col4a2** | 1.62 | 3.94 | 5.34 | 5.06 | 3.48 | 5.29 | 4.21 | 4.29 | 3.71 |
| **Col4a3** | 6.94 | 12.71 | 13.24 | 10.22 | 14.00 | 16.23 | 13.79 | 14.00 | 13.39 |
| **Col5a1** | 2.67 | 3.59 | 4.49 | 6.32 | 3.65 | 4.94 | 4.68 | 3.95 | 3.56 |
| **Col6a1** | 4.61 | 2.00 | 2.65 | 3.25 | 0.93 | 3.93 | 2.46 | 2.43 | 2.94 |
| **Ctgf** | -1.95 | 4.80 | 8.01 | 6.03 | 5.40 | 3.38 | 5.17 | 3.31 | 2.41 |
| **Ctnna1** | 1.46 | 2.51 | 2.71 | 2.49 | 2.35 | 2.61 | 2.38 | 2.11 | 1.71 |
| **Ctnna2** | 13.56 | 10.38 | 10.10 | 9.03 | 11.83 | 15.85 | 12.96 | 11.57 | 11.29 |
| **Symbol** | **MOSE-E** | **MOSE-L ADH** | **MOSE-L SPH** | **MOSE-L**  **ADH-SPH Non-Reox** | **MOSE-L ADH-SPH**  **Reox** | **MOSE-L_TICV_**  **ADH** | **MOSE-L_TICV_**  **SPH** | **MOSE- L_TICV_**  **ADH SPH**  **Non-Reox** | **MOSE- L_TICV_**  **ADH SPH**  **Reox** |
|  | **ΔCT** | **ΔCT** | **ΔCT** | **ΔCT** | **ΔCT** | **ΔCT** | **ΔCT** | **ΔCT** | **ΔCT** |
| **Ctnnb1** | 1.06 | 2.50 | 2.40 | 2.40 | 1.75 | 2.41 | 2.43 | 2.24 | 2.01 |
| **Ecm1** | 2.35 | 4.30 | 2.95 | 2.77 | 3.88 | 3.89 | 3.27 | 3.63 | 3.72 |
| **Emilin1** | 10.91 | 6.82 | 7.01 | 6.77 | 6.01 | 6.48 | 6.45 | 4.69 | 5.57 |
| **Entpd1** | 10.90 | 12.34 | 13.82 | 10.16 | 16.42 | 15.68 | 14.07 | 15.61 | 12.52 |
| **Fbln1** | 10.27 | 10.99 | 11.36 | 8.85 | 13.37 | 14.37 | 14.27 | 12.70 | 11.87 |
| **Fn1** | -1.81 | -0.46 | -0.22 | 0.48 | -1.53 | 1.56 | -1.90 | -0.38 | -1.29 |
| **Hapln1** | 13.69 | 14.49 | 15.44 | 11.67 | 15.17 | 10.79 | 2.66 | 15.63 | 13.34 |
| **Hc** | 15.36 | 13.64 | 16.20 | 12.53 | 14.69 | 8.96 | 8.15 | 9.00 | 7.48 |
| **Icam1** | 6.62 | 13.59 | 13.53 | 5.94 | 12.18 | 7.78 | 7.02 | 5.90 | 7.63 |
| **Itga2** | 9.44 | 12.08 | 14.66 | 12.72 | 14.29 | 14.13 | 12.48 | 14.53 | 12.26 |
| **Itga3** | 3.57 | 3.93 | 5.40 | 5.43 | 3.89 | 3.60 | 4.58 | 4.35 | 2.71 |
| **Itga4** | 8.08 | 16.19 | 16.79 | 14.58 | 17.31 | 13.96 | 12.57 | 14.07 | 12.26 |
| **Itga5** | 2.24 | 6.17 | 5.45 | 6.30 | 5.00 | 4.87 | 5.19 | 4.53 | 3.78 |
| **Itgae** | 13.81 | 12.05 | 13.25 | 12.48 | 11.92 | 14.70 | 14.07 | 14.21 | 12.89 |
| **Itgal** | 13.69 | 10.36 | 11.52 | 10.85 | 9.48 | 10.43 | 11.99 | 9.64 | 9.89 |
| **Itgam** | 14.61 | 13.85 | 15.00 | 11.72 | 15.31 | 15.01 | 11.33 | 17.53 | 13.23 |
| **Itgav** | 1.07 | 3.74 | 3.32 | 3.78 | 2.88 | 3.00 | 3.34 | 3.55 | 2.27 |
| **Itgax** | 15.16 | 15.01 | 16.04 | 13.33 | 15.17 | 14.01 | 12.57 | 16.01 | 13.07 |
| **Itgb1** | -0.30 | 1.98 | 2.06 | 2.64 | 1.63 | 1.77 | 2.25 | 1.84 | 1.08 |
| **Itgb2** | 8.65 | 8.65 | 7.15 | 6.37 | 8.58 | 7.19 | 6.38 | 6.60 | 6.54 |
| **Itgb3** | 4.69 | 11.78 | 10.76 | 8.91 | 11.44 | 10.20 | 8.31 | 8.91 | 10.19 |
| **Itgb4** | 6.05 | 9.32 | 9.26 | 8.90 | 8.50 | 8.10 | 6.24 | 6.94 | 7.98 |
| **Symbol** | **MOSE-E** | **MOSE-L ADH** | **MOSE-L SPH** | **MOSE-L**  **ADH-SPH Non-Reox** | **MOSE-L ADH-SPH**  **Reox** | **MOSE-L_TICV_**  **ADH** | **MOSE-L_TICV_**  **SPH** | **MOSE- L_TICV_**  **ADH SPH**  **Non-Reox** | **MOSE- L_TICV_**  **ADH SPH**  **Reox** |
|  | **ΔCT** | **ΔCT** | **ΔCT** | **ΔCT** | **ΔCT** | **ΔCT** | **ΔCT** | **ΔCT** | **ΔCT** |
| **Lama1** | 6.76 | 13.29 | 16.09 | 12.43 | 18.60 | 15.54 | 14.03 | 17.11 | 14.93 |
| **Lama2** | 11.80 | 15.17 | 15.87 | 15.51 | 17.40 | 11.89 | 14.07 | 14.69 | 14.18 |
| **Lama3** | 12.61 | 14.79 | 17.12 | 12.93 | 13.62 | 12.56 | 12.57 | 14.39 | 12.17 |
| **Lamb2** | 9.92 | 6.72 | 6.61 | 4.71 | 6.23 | 9.63 | 5.78 | 6.57 | 6.19 |
| **Lamb3** | 7.76 | 12.67 | 10.28 | 10.46 | 11.99 | 9.82 | 11.17 | 11.94 | 12.61 |
| **Lamc1** | 3.58 | 4.72 | 5.05 | 4.99 | 3.46 | 5.77 | 4.22 | 4.56 | 3.92 |
| **Mmp10** | 6.18 | 11.02 | 7.81 | 4.48 | 11.74 | 6.56 | 6.82 | 10.93 | 7.23 |
| **Mmp11** | 4.46 | 6.46 | 5.42 | 4.32 | 4.36 | 6.34 | 6.35 | 5.60 | 3.89 |
| **Mmp12** | 14.90 | 13.71 | 14.15 | 9.97 | 15.17 | 14.32 | 12.57 | 16.23 | 13.49 |
| **Mmp13** | 3.94 | 9.26 | 7.46 | 1.77 | 6.60 | 7.28 | 4.02 | 7.64 | 5.05 |
| **Mmp14** | 2.10 | 5.25 | 5.46 | 6.62 | 3.88 | 8.62 | 9.74 | 6.44 | 6.05 |
| **Mmp15** | 10.18 | 16.34 | 15.55 | 12.38 | 15.02 | 14.01 | 12.57 | 16.77 | 13.48 |
| **Mmp1a** | 6.45 | 6.69 | 8.62 | 7.79 | 8.65 | 9.14 | 9.16 | 8.85 | 7.16 |
| **Mmp2** | 2.12 | 8.35 | 9.68 | 11.40 | 8.42 | 9.90 | 10.84 | 9.68 | 9.70 |
| **Mmp3** | 5.98 | 9.32 | 6.38 | 3.82 | 9.72 | 4.13 | 4.44 | 6.60 | 4.83 |
| **Mmp7** | 15.19 | 13.72 | 15.06 | 12.42 | 15.17 | 14.10 | 12.57 | 16.94 | 13.80 |
| **Mmp8** | 13.29 | 13.96 | 11.81 | 10.29 | 12.37 | 14.14 | 11.64 | 15.17 | 11.95 |
| **Mmp9** | 6.06 | 12.19 | 12.21 | 11.25 | 12.95 | 9.73 | 8.69 | 9.37 | 8.41 |
| **Ncam1** | 1.83 | 4.14 | 3.75 | 3.63 | 2.93 | 4.32 | 1.45 | 2.89 | 2.75 |
| **Ncam2** | 13.69 | 13.94 | 15.19 | 14.22 | 15.17 | 11.58 | 12.84 | 11.10 | 11.68 |
| **Pecam1** | 14.70 | 12.65 | 13.82 | 12.23 | 12.78 | 13.94 | 12.54 | 13.65 | 12.17 |
| **Symbol** | **MOSE-E** | **MOSE-L ADH** | **MOSE-L SPH** | **MOSE-L**  **ADH-SPH Non-Reox** | **MOSE-L ADH-SPH**  **Reox** | **MOSE-L_TICV_**  **ADH** | **MOSE-L_TICV_**  **SPH** | **MOSE- L_TICV_**  **ADH SPH**  **Non-Reox** | **MOSE- L_TICV_**  **ADH SPH**  **Reox** |
|  | **ΔCT** | **ΔCT** | **ΔCT** | **ΔCT** | **ΔCT** | **ΔCT** | **ΔCT** | **ΔCT** | **ΔCT** |
| **Postn** | 10.69 | 16.09 | 18.23 | 11.67 | 15.17 | 15.01 | 2.57 | 17.13 | 13.46 |
| **Sele** | 12.70 | 13.24 | 14.00 | 11.78 | 15.17 | 14.18 | 14.26 | 14.09 | 12.72 |
| **Sell** | 9.49 | 14.32 | 11.90 | 13.18 | 15.17 | 15.01 | 14.06 | 17.40 | 15.64 |
| **Selp** | 9.74 | 7.16 | 5.45 | 7.53 | 6.65 | 9.67 | 10.08 | 9.72 | 9.67 |
| **Sgce** | 4.53 | 14.01 | 19.14 | 11.81 | 14.96 | 14.01 | 12.57 | 15.76 | 13.35 |
| **Sparc** | -2.32 | -0.87 | 0.40 | 0.89 | -0.67 | 0.67 | 0.52 | -0.25 | -0.79 |
| **Spock1** | 14.39 | 13.47 | 17.40 | 12.32 | 13.14 | 14.01 | 14.43 | 16.83 | 15.48 |
| **Spp1** | -0.28 | 0.62 | 1.73 | 0.60 | 2.06 | -2.18 | 0.00 | -0.90 | -1.70 |
| **Syt1** | 13.51 | 13.34 | 14.84 | 12.28 | 13.35 | 13.35 | 12.57 | 15.09 | 13.28 |
| **Tgfbi** | 12.37 | 11.69 | 12.13 | 9.69 | 11.18 | 15.76 | 12.10 | 15.30 | 13.39 |
| **Thbs1** | 0.04 | 3.36 | 3.29 | 3.86 | 2.64 | 1.81 | 2.51 | 1.91 | 1.52 |
| **Thbs2** | 10.55 | 14.24 | 17.23 | 14.33 | 15.17 | 7.68 | 6.53 | 5.45 | 8.41 |
| **Thbs3** | 8.37 | 5.96 | 7.65 | 9.62 | 6.24 | 8.58 | 9.12 | 8.60 | 8.55 |
| **Timp1** | 4.38 | 4.96 | 7.17 | 6.80 | 5.68 | 5.23 | 5.99 | 6.19 | 4.08 |
| **Timp2** | -0.68 | 1.29 | 2.37 | 1.11 | 0.98 | 1.19 | 2.57 | 1.95 | 0.71 |
| **Timp3** | -0.66 | 0.97 | 2.65 | 2.54 | 0.17 | 2.03 | 1.64 | 1.39 | 0.62 |
| **Tnc** | 1.94 | 3.28 | 5.87 | 3.90 | 2.65 | 8.12 | 4.03 | 6.04 | 4.41 |
| **Vcam1** | 0.17 | 3.69 | 3.79 | 2.59 | 2.37 | 5.08 | 4.75 | 5.41 | 4.33 |
| **Vcan** | 4.23 | 3.51 | 2.99 | 5.69 | 3.05 | 3.37 | 3.89 | 3.55 | 3.27 |
| **Vtn** | 15.21 | 13.97 | 15.08 | 12.39 | 14.46 | 14.01 | 12.57 | 15.69 | 13.34 |

**Table S2.** **Gene expression of adhesion molecules, extracellular matrix (ECM) and their regulators. Δ**CT changes during MOSE progression and between adherent cells (ADH), aggregation (SPH) and adherent spheroid stage before (ADH SPH NON-REOX) and after reoxygenation (ADH SPH REOX).
