# Supplementary material for: Dynamic integrin expression, atypical nuclear localization, and spatial distribution during ovarian cancer progression and metastasis
Source: Front Cell Dev Biol. 2026 Feb 25;14:1744403. doi: 10.3389/fcell.2026.1744403 (PMC12975958; doi:10.3389/fcell.2026.1744403)
Supplement: Supplementary file 3 [file Table3.docx]

Table S3

| Drug | Purchased | What they are | What do they do |
| --- | --- | --- | --- |
| BT3033 | Tocris Bioscience | BTT-3033 is a synthetic, fluorinated, sulfonamide-containing aromatic urea with a heterocyclic pyrazole core. | BTT 3033 is a selective inhibitor of integrin α_2_β_1_. It binds to the α_2_I domain. Exhibits selectivity for α_2_β_1_ over integrins α_3_β_1_, α_4_β_1_, α_5_β_1_ and α_v_. |
| E7820 | Tocris Bioscience | E7820 is a synthetic small-molecule aromatic sulfonamide derivative studied primarily as an anti-angiogenic and anticancer agent | E7820 key mechanism is suppressing the expression of integrin α2 on endothelial and some cancer cells |
| αvβ1 integrin-IN-1 TFA(Compound C8) | MedChemExpress | αvβ1 integrin-IN-1 (also known as Compound C8) is a synthetic small-molecule inhibitor developed for research purposes to selectively block the activity of the αvβ1 integrin. | Compound C8 acts by directly antagonizing αvβ1 integrin at the extracellular ligand-binding site. By occupying this binding pocket, the compound prevents αvβ1 from engaging its ECM ligands, thereby disrupting integrin-mediated adhesion and signaling. Importantly, this mechanism does not involve changes in integrin expression or protein degradation; instead, it blocks integrin function at the cell surface. Functionally, inhibition of αvβ1 with Compound C8 leads to reduced cell adhesion and spreading, impaired formation of mature focal adhesions, and diminished transmission of mechanical forces to the ECM |
| GLPG0187 | Tocris Bioscience | Chemically, GLPG0187 is a synthetic, low–molecular-weight, non-peptide small molecule designed to act as an RGD-mimetic integrin antagonist. | GLPG0187 works by directly blocking the ligand-binding function of αv integrins at the cell surface. It is chemically designed to act as an RGD mimetic, meaning it imitates the structural features of the Arg-Gly-Asp (RGD) motif that many extracellular matrix (ECM) proteins use to bind αv integrins. By mimicking this motif, GLPG0187 competes with natural ECM ligands for access to the integrin binding site. |
| TC-I 15 | Tocris Bioscience | TC-I 15 is a synthetic, heterocyclic small molecule with specific functional groups designed to selectively target collagen-binding integrins. | The compound acts as an allosteric antagonist, binding to the integrin and stabilizing it in an inactive conformation, thereby preventing its interaction with collagen in the extracellular matrix. |
| AIIB2 | Developmental Studies Hybridoma Bank (DSHB) at the University of Iowa. | monoclonal IgG1 | AIIB2 blocking antibody targets the β1 integrin subunit and functions by allosterically stabilizing its bent, low-affinity conformation, thereby inhibiting its function |
| Cyclo (RGDyK) | MedChemExpress | Cyclo (RGDyK) inhibitor is a potent, selective peptide drug candidate targeting the αvβ3 integrin | Cyclo (RGDyK) binds to αvβ3 integrin, competitively blocking its interaction with natural RGD-containing ligands in the extracellular matrix. By doing so, it inhibits integrin-mediated cell adhesion |
| K34c | Tocris Bioscience | K34c is a chemically engineered, heterocyclic small molecule designed to selectively inhibit α5β1 integrin function in cellular and preclinical studies. | K34c acts as a potent antagonist of the α5β1 integrin, meaning it binds to this receptor and blocks its normal interaction with extracellular matrix proteins like fibronectin. This blockade interferes with integrin-mediated cell adhesion and migration |
