# Supplementary figures and images for: Dynamic integrin expression, atypical nuclear localization, and spatial distribution during ovarian cancer progression and metastasis
Source: Front Cell Dev Biol. 2026 Feb 25;14:1744403. doi: 10.3389/fcell.2026.1744403 (PMC12975958; doi:10.3389/fcell.2026.1744403)

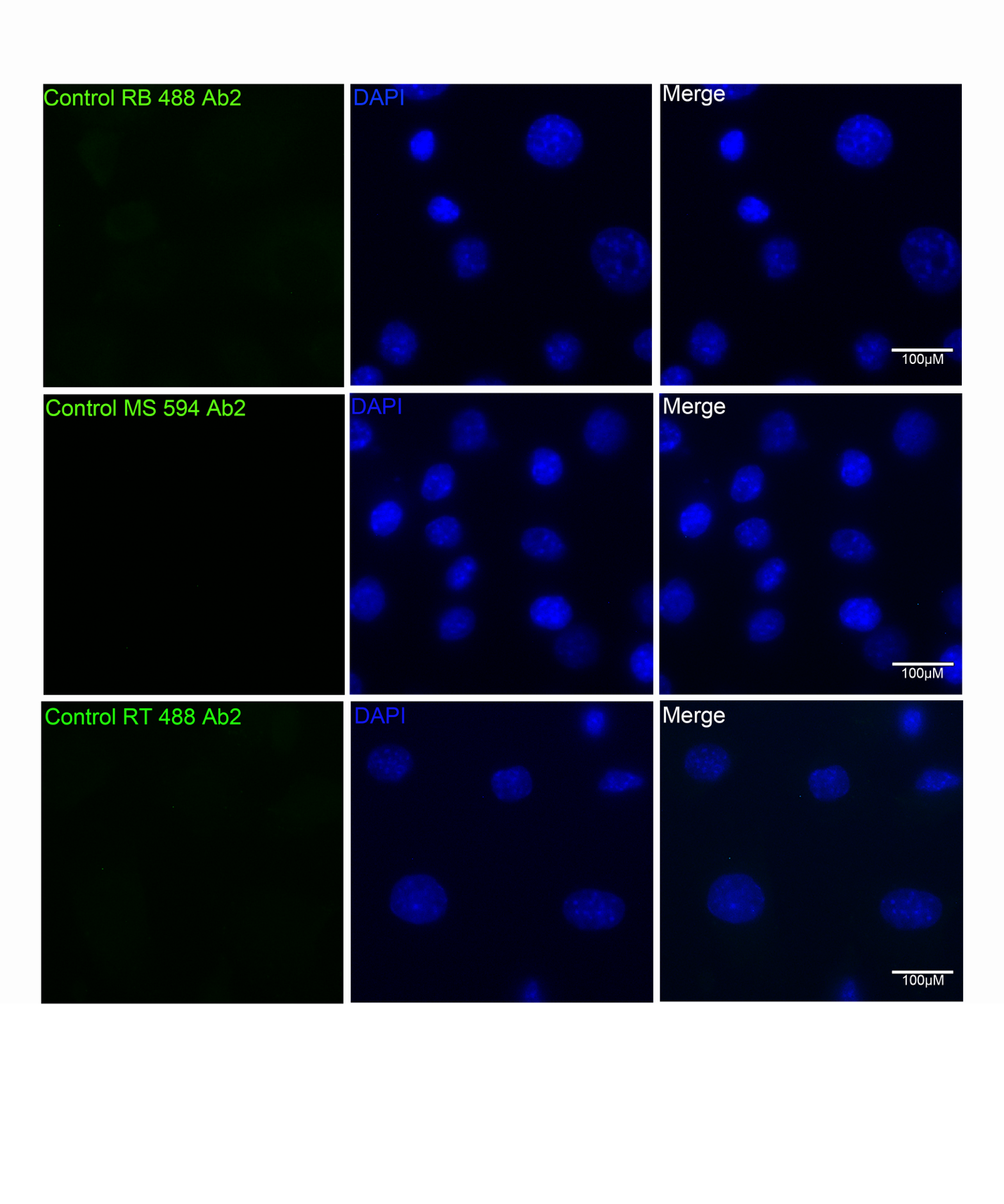


Supplemental Figure S1

Supplement: Supplementary file 5 [file DataSheet1.docx]
